# Identification and characterization of non-canonical azole antifungal resistance pathways in *Aspergillus fumigatus*

**DOI:** 10.1128/mbio.01723-26

**Published:** 2026-08-19

**Authors:** Endrews Delbaje, Matheus Mertz Ribeiro, Ricardo de Oliveira Barbosa Bitencourt, Katrien Lagrou, Vit Hubka, Laís Pontes, Angélica Zaninelli Schreiber, Nicole Robbins, Leah E. Cowen, Thaila Fernanda dos Reis, Gustavo H. Goldman

**Affiliations:** 1 Departamento de Biologia, Faculdade de Filosofia, Ciências e Letras de Ribeirão Preto, Universidade de São Paulo 124588, Ribeirao Preto, São Paulo, Brazil; 2 Faculdade de Ciências Farmacêuticas de Ribeirão Preto, Universidade de São Paulo 67782, Ribeirao Preto, São Paulo, Brazil; 3 Department of Laboratory Medicine, National Reference Centre for Mycosis, University Hospitals Leuven 60182 , Leuven, Belgium; 4 Department of Microbiology, Immunology and Transplantation, Laboratory of Clinical Microbiology, KU Leuven 573654, Leuven, Belgium; 5 Department of Genetics and Microbiology, Faculty of Science, Charles University 112302, Prague, Czech Republic; 6 Laboratory of Fungal Genetics and Metabolism, Institute of Microbiology, Czech Academy of Sciences 48311, Prague, Czech Republic; 7 School of Medical Sciences, University of Campinas 67791, Campinas, São Paulo, Brazil; 8 Department of Molecular Genetics, University of Toronto 204248, Toronto, Ontario, Canada; 9 Departamento de Bioquímica, Instituto de Química, Universidade Estadual de São Paulo, Araraquara, Brazil; 10 National Institute of Science and Technology in Human Pathogenic Fungi, Ribeirão Preto, Sao Paulo, Brazil; Universiteit Gent, Gent, Belgium

**Keywords:** azole resistance, *Aspergillus fumigatus*, evolution, Cyp51, noncanonical Cyp51 resistance

## Abstract

Human fungal infections, especially those caused by *Aspergillus fumigatus*, pose a significant global health threat, particularly in immunocompromised individuals. Azole antifungals are the primary treatment for this pathogen; however, the prevalence of azole-resistant *A. fumigatus* strains is steadily increasing. Mutations in *cyp51A*, which encodes an enzyme involved in ergosterol biosynthesis and the molecular target of the azoles, are well established to confer resistance in this fungal species. However, additional mechanisms governing resistance to this antifungal class remain understudied and poorly characterized, despite growing recognition of their importance in clinical resistance. In this study, we investigated the genetic basis of azole resistance in *A. fumigatus* isolates from clinical settings worldwide, with a particular focus on mechanisms independent of *cyp51A* (non-canonical). Using a combination of genomic and functional approaches, including whole-genome sequencing and transcriptomic analysis, we identified novel genetic variants and characterized population structure, advancing our understanding of the genetic diversity and evolutionary dynamics of resistance in *A. fumigatus*. By expanding our understanding of the complex genetic and molecular factors underlying azole resistance in this important human fungal pathogen, this research is poised to inform the development of novel antifungal strategies and contribute to global efforts to combat fungal infections.

**IMPORTANCE:**

Azole antifungals are the frontline therapy for infections caused by the opportunistic mold *Aspergillus fumigatus*, yet resistance to these drugs is rapidly increasing worldwide. Most studies have focused on mutations in *cyp51A*, the canonical target of azoles; however, a growing proportion of resistant clinical isolates lack these mutations, indicating that alternative resistance mechanisms are emerging. Here, we integrate population genomics, transcriptomics, and functional analyses across a global collection of isolates to define the architecture of *cyp51*-independent (non-canonical) azole resistance. We show that this resistance phenotype is strongly associated with a distinct population lineage and is driven by a highly polygenic network of metabolic, mitochondrial, and regulatory adaptations rather than single target site mutations. These isolates exhibit extensive transcriptional rewiring and metabolic remodeling under azole stress, suggesting distinct survival strategies beyond canonical resistance. Our findings reveal that azole resistance in *A. fumigatus* can evolve through diverse evolutionary routes and emphasize the need to monitor and therapeutically target non-canonical pathways that may increasingly contribute to antifungal treatment failure.

## INTRODUCTION

Fungal infections in humans are a growing public health concern. Over the past 30 years, multiple factors have contributed to a dramatic increase in fatal fungal infection cases, driven by the growth of immunocompromised populations. Some of these factors include the following: advances in organ transplantation, which require the use of immunosuppressive drugs (1); cancer treatments that can weaken the immune system (2); the HIV/AIDS epidemic (3); and increased humidity and temperature due to climate change, creating more favorable conditions for fungal growth (4). An estimated 2.1 million cases of invasive aspergillosis occur annually, contributing to a global burden of over 6–7 million invasive fungal infections and approximately 2.5 million deaths directly attributable to fungal disease (5). *Aspergillus fumigatus* is an opportunistic pathogen and the primary *Aspergillus* species responsible for aspergillosis, including invasive pulmonary aspergillosis (IPA) cases (6), and is a significant cause of morbidity and mortality worldwide (5). This led to its recent inclusion on the World Health Organization’s (WHO) Fungal Priority Pathogens List (7). Furthermore, *A. fumigatus* is a ubiquitous species found mainly in soil, with a global distribution (8).

Azoles are a class of antifungal drugs widely used in both hospitals and agriculture (9). For *A. fumigatus*, the main azoles used for treatment include voriconazole, isavuconazole, itraconazole, and posaconazole (10). These molecules target the fungal cytochrome P450 enzyme Cyp51A, inhibiting ergosterol biosynthesis, a crucial component of the fungal cell membrane (11). When *A. fumigatus* is exposed to azoles, it can adjust its physiology to survive, allowing genetic adaptations to emerge through natural selection. Its ability to acquire resistance in patients during treatments has been demonstrated (12, 13), and the widespread use of azole fungicides in agriculture has been linked to the emergence of resistance in environmental isolates (11). Alarmingly, these environmentally selected resistant strains have also been implicated in contributing to clinical resistance in patients with no prior azole exposure.

One of the best-characterized azole resistance mechanisms in *A. fumigatus* involves two linked genetic mutations in *cyp51A*: TR34/L98H. This results in a 34-base-pair tandem repeat in the promoter region of the gene, leading to overexpression, coupled with a leucine-to-histidine substitution in the enzyme that reduces azole binding (11). Despite the importance of this combination of mutations in conferring azole resistance, recent studies have highlighted the emergence of alternative mechanisms of azole resistance. These mechanisms often involve complex genetic and regulatory networks, making them more difficult to understand (14–16). Thus, despite their relevance, these uninvestigated mutations, independent of *cyp51A*, likely influence key cellular processes that lead to resistance.

Understanding the mechanisms underlying azole resistance in clinical and environmental isolates of *A. fumigatus* is essential for developing effective antifungal strategies. By investigating the genetic and physiological adaptations associated with resistance, we can identify potential novel targets for new antifungal therapies. Additionally, studying the impact of environmental azole exposure on the evolution of resistance can inform public health policies to mitigate the spread of resistant lineages. In this context, we aimed to investigate the genomic basis of azole resistance in *A. fumigatus* isolates from clinical and environmental settings worldwide, focusing on mechanisms independent of the well-characterized *cyp51A* mutations. By focusing on such novel resistance mechanisms, we identified new pathways and genetic variations that could possibly contribute to resistance.

## RESULTS

### Genome sequencing, phylogenetic analysis, and resistance classification of *A. fumigatus* isolates

To investigate azole resistance mechanisms beyond those governed by canonical *cyp51A* mutations, we first identified and sequenced the genomes of 26 clinical *A. fumigatus* isolates that lacked known mutations in the *cyp51A* promoter and coding region (Table 1). We also sequenced the genomes of 10 additional clinical isolates harboring mutations in *cyp51A* that have not been previously described to confer azole resistance (Table 1). BUSCO analysis indicated high genome completeness, with an average of 98.81% (range: 96.2–99.1%). The assembled genomes had a mean size of 28.663 Mb (range: 26.222–30.020 Mb) and were sequenced to an average coverage of 90.19× (range: 13.46–426.99×). These 36 newly sequenced genomes were combined with 334 publicly available genomes, bringing our total data set of *A. fumigatus* genome sequences to 370. To classify each strain as azole-resistant or susceptible, we performed susceptibility testing on the 36 newly sequenced isolates and used data from the original publications for public genomes. Isolates were classified as resistant if the minimum inhibitory concentration (MIC) for any of the triazoles tested—itraconazole (ITC), voriconazole (VOR), or posaconazole (POS)—exceeded the threshold (as detailed in Materials and Methods). This resulted in the resistance profiles of these 370 isolates being categorized as follows: 179 azole-susceptible, 125 azole-resistant with known *cyp51A* mutations*,* and 66 azole-resistant via presumed non-canonical mechanisms. More specifically, the strains included 173 ITC-resistant, 132 VOR-resistant, and 61 POS-resistant isolates (see Table S1 at https://doi.org/10.6084/m9.figshare.32975738). Isolates originated from diverse geographical regions, including Belgium (20), Brazil (9), China (27), the Czech Republic (7), India (8), Japan (70), the Netherlands (8), Spain (4), and the United Kingdom (216) (see Table S1 at https://doi.org/10.6084/m9.figshare.32975738).

**TABLE 1:** List of strains used in this work^*b*^

Strain	MIC/MEC (µg/mL)						Clinical material	Origin	Resistance^*a*^	Canonical or non-canonical	*cyp51A* resistance mutation
	VOR	CAS	ISA	AMP	POS	ITRA					
A1163	0.5	0.007	0.5	0.25	0.03	0.25	ND	ND	It is susceptible to azoles	Susceptible	
CYP108	2	0.03	2	1	1	0.5	ND	KL	POS	*cyp51A* independent	
CYP109	0.5	0.03	≥4	1	2	≥4	BAL	KL	ITRA	*cyp51A* independent	
CYP134	2	0.03	≥4	1	2	1	Respiratory specimen from a patient with cystic fibrosis	KL	ISAVU	*cyp51A* independent	
CYP146	1	1.6	1	1	1	1	Respiratory specimen from a patient with cystic fibrosis	KL	ITRA and POS	*cyp51A* independent	
CYP147	4	0.03	≥4	2	2	≥4	BAL	KL	VOR, POS, and ITRA	*cyp51A* dependent	TR34
CYP171	4	0.03	≥4	1	1	0.25	BAL	KL	VOR, ITRA, and ISAVU	*cyp51A* dependent	TR34
CYP180	4	0.03	≥4	2	2	≥4	Respiratory specimen from a patient with cystic fibrosis	KL	VOR, POS, ITRA, and ISAVU	*cyp51A* independent	
CYP184	4	0.03	≥4	1	2	≥4		KL	VOR, POS, ITRA, and ISAVU	*cyp51A* dependent	Leu98His, TR34
CYP212	≥4	0.03	≥4	2	2	≥4	Respiratory specimen from a patient with cystic fibrosis	KL	VOR, POS, ITRA, and ISAVU	*cyp51A* dependent	TR34
CYP215	2	0.03	≥4	2	2	1	ND	KL	ISAVU and POS	*cyp51A* dependent	TR34
CYP224	≥4	0.03	≥4	1	4	≥4	ND	KL	VOR, ISAVU, ITRA, and POS	*cyp51A* dependent	Tyr121Phe, Thr289Ala
CYP225	≥4	0.03	≥4	2	2	≥4	ND	KL	VOR, ISAVU, ITRA, and POS	*cyp51A* independent	
CYP256	2	0.03	≥4	1	2	≥4	Biopsy/tissue (lung)	KL	ISAVU	*cyp51A* independent	
CYP262	≥4	0.03	≥4	1	2	1	Bronchial aspirate	KL	VOR, POS, ITRA, and ISAVU	*cyp51A* dependent	Tyr121Phe, Gly448Ser, TR34
CYP263	≥4	0.007	≥4	1	2	≥4	BAL	KL	VOR, POS, and ISAVU	*cyp51A* independent	
CYP297	4	0.03	≥4	1	1	≥4	Sputum	KL	VOR, ITRA, and ISAVU	*cyp51A* dependent	Leu98His, TR34
CYP300	2	1	≥4	1	2	2	Bronchial aspirate	KL	VOR, POS, ITRA, and ISAVU	*cyp51A* dependent	Leu98His, TR34
CYP312	2	ND	2	0.25	0.13	>16	Biopsy/tissue (lung)	KL	VOR, POS, ITRA, and ISAVU	*cyp51A* dependent	Leu98His, TR34
CYP315	1	1	≥4	2	1	2	BAL	KL	VOR and ISAVU	*cyp51A* independent	
CYP322	1	0.7	4	2	1	0.5	Respiratory specimen from a patient with cystic fibrosis	KL	ISAVU	*cyp51A* independent	
LIF2328	≥4	0.03	≥4	2	1	≥4	Sputum	AS	VOR, ITRA, ISAVU, and POS	*cyp51A* independent	
LIF23A	≥4	0.007	1	2	0.5	≥4	Sputum	AS	VOR and ISAVU	*cyp51A* independent	
LIF472	1	0.007	2	2	1	≥4	Sputum	AS	ITRA and POS	*cyp51A* independent	
LIF479	1	0.015	2	2	1	≥4	Sputum	AS	ITRA and POS	*cyp51A* independent	
LIF536	1	0.015	2	2	1	≥4	Sputum	AS	ITRA and POS	*cyp51A* independent	
LIF543	≥4	0.015	≥4	2	2	≥4	Oropharyngeal secretion	AS	VOR, ITRA, ISAVU, and POS	*cyp51A* independent	
LIF621	1	0.015	2	2	1	≥4	Sputum	AS	ISAVU and POS	*cyp51A* independent	
LIF710	1	0.03	2	2	1	≥4	Sputum	AS	ISAVU and POS	*cyp51A* independent	
LIF737	1	0.015	1	2	1	1	BAL	AS	POS	*cyp51A* independent	
CCF6650	1	0.03	≥4	1	1	≥4	Sputum	VH	VOR, ITRA, ISAVU, and POS	*cyp51A* independent	
CCF6677	2	0.007	4	1	0.5	≥4	Swab from wound	VH	ITRA and ISAVU	*cyp51A* independent	
CCF6440	2	0.007	≥4	1	0.5	≥4	BAL	VH	VOR, ITRA, and ISAVU	*cyp51A* independent	
CCF6739	≥4	0.007	2	2	4	≥4	Bronchial aspirate	VH	VOR, ISAVU, and POS	*cyp51A* independent	
CCF6740	1	0.007	2	1	0.25	≥4	BAL	VH	ND	*cyp51A* independent	
CCF6741	1	0.007	2	1	0.5	≥4	Ear swab	VH	VOR	*cyp51A* independent	
CCF6738	1	0.007	2	1	0.5	≥4	Bronchial aspirate	VH	ND	*cyp51A* independent	

^*a*^ According to cutoff values mentioned in Materials and Methods.

^*b*^ KL, laboratory of Katrien Lagrou, Belgium; AS, laboratory of Angélica Schreiber, Brazil; VH, laboratory of Vit Hubka, Czech Republic; ND, not determined; BAL, roncoalveolar lavage.

**Fig 1:**
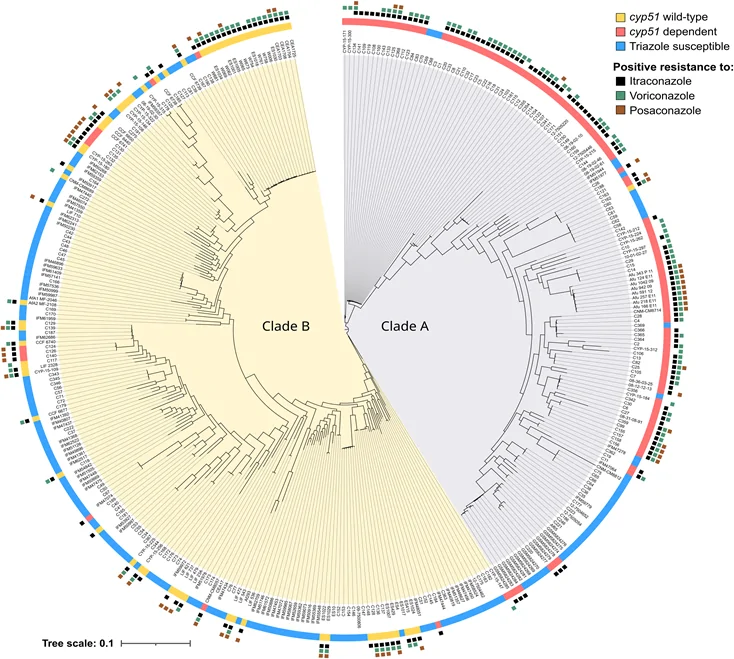
Phylogenetic analysis based on SNPs. Maximum-likelihood phylogeny of 370 *A. fumigatus* genomes, colored by resistance phenotype. Resistance to specific azole drugs is annotated by colored squares on the outer ring. The two predefined major clades, A and B, are highlighted in light yellow and light gray, respectively. Scale bar (0.1) represents the number of substitutions per site.

The phylogenetic reconstruction of the 370 *A. fumigatus* genomes revealed the well-established major clades A and B (17). Interestingly, distinct distributions of VOR resistance types were noted across the clades. Clade A (*n* = 158) was significantly enriched for isolates with canonical mutations that confer azole resistance (114/158 isolates) and also included 43 susceptible isolates and a single *cyp51*-independent resistant strain (Fig. 1). Clade B (*n* = 212) was the primary reservoir of *cyp51*-independent resistant strains, accounting for 65 of the 66 such isolates (Fig. 1). This clade also contained 134 susceptible and 13 *cyp51*-dependent isolates. This phylogenetic partitioning provides genomic evidence that *cyp51-*dependent and *cyp51*-independent resistance mechanisms may have emerged from separate evolutionary trajectories, and that lineage-specific genetic backgrounds in *A. fumigatus* may predispose isolates to develop one resistance pathway rather than the other.

### Pangenome and accessory resistance factors

Analysis of the *A. fumigatus* pangenome across 370 isolates identified 11,268 orthogroups, with a core genome of 8,567 genes (7,878 single-copy genes). The accessory genome, comprising 2,701 genes, represents a significant source of functional diversity and is often enriched for genes involved in environmental niche adaptation, such as carbohydrate-active enzymes and secondary metabolite biosynthetic gene clusters (BGCs) (see Table S2 at https://doi.org/10.6084/m9.figshare.32975738). SCOARY analysis identified 291 orthogroups significantly associated with *cyp51*-independent resistance (Bonferroni *P* < 0.05) (see Table S2 at https://doi.org/10.6084/m9.figshare.32975738). While many of these are genes of unknown function, several significant associations were found in co-localized genomic clusters, indicating that the acquisition or retention of specific metabolic modules may drive non-canonical resistance. The top associations (eight most significant) are from genes of unknown function. Among the associated orthogroups with functional identification, OG0009309 is identified as a mitochondrial carrier protein (Pet8), which is more commonly found in clade B than in clade A (see Table S2 at https://doi.org/10.6084/m9.figshare.32975738). It was also associated with canonical and non-canonical azole resistance, being enriched in *cyp51*-independent isolates and decreased in *cyp51*-dependent isolates. A few additional important associations occurred in clustered genes, as exemplified by the genes clustered around the *cyp52* gene, with orthogroup OG0010061 represented by CYP52 (AFUA_5G00120: cytochrome P450 oxidoreductase/alkane hydroxylase), OG0010062 (squalene-hopene-cyclase), OG0010063 (AFUA_5G00100: integral membrane protein), OG0010059 (AFUA_5G00135: C6 finger domain protein), and OG0010060 (AFUA_5G00130: capsule polysaccharide biosynthesis protein).

### Non-azole fungicide resistance mutations are enriched in clade A and co-occur with canonical azole resistance

To explore whether environmental fungicide exposure could be inferred from our collection, we screened all 370 isolates for mutations in *benA* (encoding β-tubulin, conferring benzimidazole resistance) and *sdhB* (succinate dehydrogenase subunit B, conferring SDHI resistance) (18) (see Table S1 at https://doi.org/10.6084/m9.figshare.32975738). A total of 48 isolates carried mutations in these genes (48 in *benA*, seven in *sdhB*). Of these, 44 (91.7%) belonged to clade A, and 43 were azole-resistant exclusively through cyp51A-dependent mechanisms (canonical resistance). Among the *benA*/*sdhB*-positive isolates, 35 harbored tandem-repeat (TR) mutations in the *cyp51A* promoter. Notably, only five isolates carrying non-azole resistance mutations were azole susceptible, and none were found among the non-canonical resistant group.

### Genomic differentiation and evolutionary dynamics reveal signatures of selection

**Fig 2:**
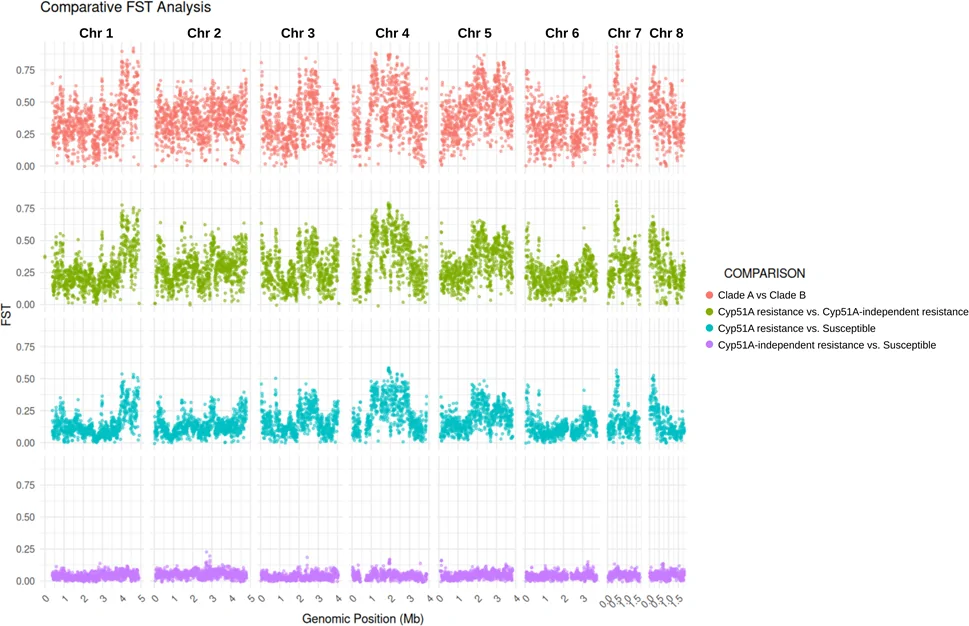
Scatterplot of sliding 5 kb non-overlapping window estimates of FST for each chromosome between isolates comparing clades A and B, Cyp51A resistance groups, and susceptible isolates. The dots representing genomic windows are colored based on the presence of mutations significantly associated with azole resistance.

Fixation index (FST) analysis provides a quantitative measure of genetic differentiation between populations, and its application to the 370-genome data set revealed that selection likely acts on these clades. The highest observed FST values across genome windows were observed when comparing clade A against clade B (Fig. 2), showing that this classification is the primary source of genetic variance in the species. Comparisons between *cyp51*-dependent and *cyp51*-independent resistant groups also showed high FST values, reflecting deep lineage divergence between their respective clades (A and B) rather than solely the resistance phenotype (Fig. 2). In contrast, low FST values were observed between *cyp51*-independent resistant and azole-susceptible isolates within clade B, suggesting that the transition from susceptibility to resistance in this non-canonical resistance group does not involve a large genomic shift or a single hard sweep of a dominant genotype. Instead, it suggests a polygenic basis in which resistance is conferred by combinations of SNPs and accessory genes that may already be present as standing genetic variation within the susceptible population.

To evaluate the temporal and selective context of the differentiation between clades A and B, Tajima’s *D* was calculated for genome windows across both lineages. The analysis intersected high-FST peaks (top 10%) with Tajima’s *D* results (see Table S3 at https://doi.org/10.6084/m9.figshare.32975738), revealing a strong signature of recent selection at the very sites that define clade divergence. Of the 254 windows localized to high FST peaks, 97.6% (248) had negative Tajima’s *D* values in clade B, and 83.5% (212) had negative values in clade A. This confirms that these genomic regions, despite being from an old divergence, are under active selective pressure in the contemporary population.

### Phenotypic characterization of representative azole-resistant clinical isolates

To examine phenotypic differences among azole-resistant strains, we selected 11 azole-resistant clinical isolates (four cyp51-dependent: CYP147, CYP184, CYP212, LIF3546; and seven cyp51-independent: CCF6440, CYP180, CYP225, CYP256, CYP263, CYP322, and LIF543) and compared them with the azole-susceptible reference strain A1160 *pyrG*^+^. When cultured on minimal medium (MM) at 37°C for 5 days in the absence of any stressor, these strains showed comparable radial growth, suggesting that any mutations acquired to confer azole resistance did not impose a fitness cost (see Fig. S1 at https://doi.org/10.6084/m9.figshare.32975738). We then assessed whether any of the isolates showed differences in susceptibility to diverse stressors, including oxidative stressors such as *t*-butyl hydroperoxide and hydrogen peroxide, as well as antifungal agents such as amphotericin B and miltefosine. Slight differences in susceptibility were noted among isolates: strains CYP212, CYP184, CYP180, CYP322, CYP263, and CCF6440 showed twofold greater resistance to t-butyl hydroperoxide, while CYP212, LIF3546, and CCF6440 showed twofold greater resistance to hydrogen peroxide. In addition, two strains, CYP184 and CCF6440, showed twofold greater resistance to amphotericin than the reference strain A1160 *pyrG*^+^ (see Table S4 at https://doi.org/10.6084/m9.figshare.32975738). None of the strains showed any differential susceptibility to the antifungal and anti-leishmanial drug miltefosine (see Table S4 at https://doi.org/10.6084/m9.figshare.32975738). Importantly, no overall trends were noted when comparing the phenotypes of *cyp51-*dependent versus *cyp51*-independent isolates.

### Transcriptional profiling of selected azole-resistant clinical isolates

**Fig 3:**
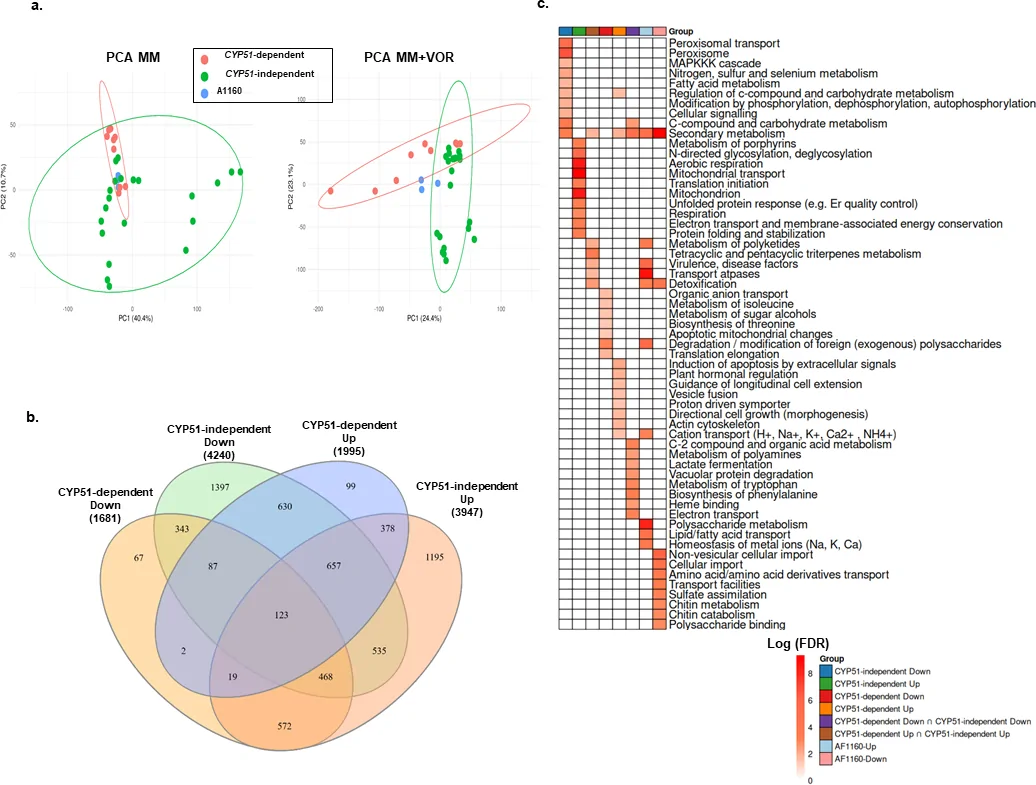
Global gene expression profiles in *A. fumigatus* isolates with distinct VOR resistance mechanisms. (**a**) Principal component analysis (PCA) of isolates grown in minimal medium (MM) control condition without and with VOR. (**b**) Venn diagram of the genes differentially expressed in the *cyp51*-dependent and *cyp51*-independent clinical isolates. (**c**) Funcat (functional categorization) of VOR-susceptible (A1160 *pyrG*^+^) and *cyp51*-dependent and *cyp51*-independent differentially expressed genes.

Given that no stark phenotypic differences were observed between *cyp51*-dependent and *cyp51*-independent lineages, we sought to determine whether these strains exhibited differences in their global transcriptional profiles. To do so, we performed RNA-Seq on the same panel of strains evaluated in see Fig. S1 at (https://doi.org/10.6084/m9.figshare.32975738). These strains were grown in MM in the absence or presence of 0.5× MIC of VOR for 24 h at 37°C, except for CYP322, which was grown for 48 h. We focused on differentially expressed genes (DEGs) with log2 fold changes ≥ 1 or ≤ −1 (see Table S5 at https://doi.org/10.6084/m9.figshare.32975738). Principal component analysis (PCA) of the global transcriptomic profiles revealed distinct patterns of separation between VOR-susceptible and VOR-resistant *A. fumigatus* isolates, which were influenced by the presence of the drug (Fig. 3a). In MM, A1160 *pyrG*^+^ and *cyp51*-dependent isolates showed considerable overlap, whereas the *cyp51-*independent resistant group exhibited a markedly heterogeneous profile along PC1, accounting for a high proportion (40.4%) of the total variance (Fig. 3a). Upon exposure to VOR, a separation emerged, with the two resistance types showing a slightly segregated pattern, while the susceptible A1160 *pyrG*^+^ reference exhibited no overlap (Fig. 3a). This suggests that VOR exposure elicits divergent and specific transcriptional responses in the two resistant types that do not occur in the reference azole-susceptible strain.

When examining the specific changes in global transcriptional responses, we identified hundreds of differentially expressed genes (DEGs) from each of the groups upon VOR treatment: A1160 *pyrG*^+^ (434 and 545 up- and downregulated), CYP147 (1,264 and 1,143 up- and downregulated), CYP212 (475 and 228 up- and downregulated), LIF3545 (235 and 378 up- and downregulated), CCF6440 (824 and 1,195 up- and downregulated), CYP180 (1,992 and 2,266 up- and downregulated), CYP225 (400 and 317 up- and downregulated), CYP256 (757 and 584 up- and downregulated), CYP263 (1,245 and 1,902 up- and downregulated), CYP322 (531 and 1,268 up- and downregulated), and LIF543 (1,015 and 1,593 up- and downregulated) (see Table S5 at https://doi.org/10.6084/m9.figshare.32975738). When comparing differential expression profiles between *cyp51-*dependent versus *cyp51*-independent groups, we identified 1,397 unique genes downregulated in the *cyp51*-independent strains, 1,195 unique genes upregulated in the *cyp51-*independent strains, 67 unique genes downregulated in the *cyp51*-dependent strains, 99 unique genes upregulated in the *cyp51-*dependent strain, 343 commonly downregulated genes, and 378 commonly upregulated genes (Fig. 3b; see Table S6 at https://doi.org/10.6084/m9.figshare.32975738). Thus, while we identified a core set of DEGs modulated in response to azoles, unique transcriptional profiles certainly exist among *A. fumigatus* strains, as reflected by the presence of *cyp51A* mutations.

We categorized the DEGs from these three classes of isolates (VOR susceptible, *cyp51* dependent resistant, *cyp51* independent resistant) using FunCat (functional categorization) and overrepresentation analysis (ORA) (https://fungifun3.hki-jena.de/ [19]). Functional categorization using the FunCat database identified significantly enriched biological pathways among the DEGs in the VOR-susceptible *A. fumigatus* strain A1160 *pyrG*^+^ following VOR exposure. Specifically, there was a concerted downregulation of primary metabolic and transport functions, contrasted by an upregulation of pathways associated with stress response, virulence, and the metabolism of complex compounds (Fig. 3c; see Table S5 at https://doi.org/10.6084/m9.figshare.32975738). Thus, VOR exposure triggers a shift in cellular priorities, suppressing primary metabolic uptake while activating biosynthetic, efflux, and virulence-associated pathways to mitigate drug-induced stress in the susceptible A1160 *pyrG*^+^ isolate.

Categorization of the 343 common downregulated genes in both *cyp51*-dependent and *cyp51*-independent strains revealed a strong metabolic shift toward pathways involved in alternative energy generation and redox balance. This includes pronounced enrichment in lactate fermentation and the electron transport chain, indicating decreased anaerobic or hypoxic metabolism and increased respiratory capacity. Concurrently, the significant enrichment in heme binding supports the heightened need for electron transport components (Fig. 3c; see Tables S5 and S6 at https://doi.org/10.6084/m9.figshare.32975738). The enrichment in secondary metabolism remained a consistent feature, underscoring its importance in the resistance phenotype (Fig. 3c; see Tables S5 and S6 at https://doi.org/10.6084/m9.figshare.32975738). Additionally, pathways such as polyamine metabolism and vacuolar protein degradation indicate adaptations in cellular stress management and proteostasis (Fig. 3c; see Tables S5 and S6 at https://doi.org/10.6084/m9.figshare.32975738).

Categorization of the 378 common upregulated genes in both *cyp51*-dependent and *cyp51*-independent strains showed secondary metabolism, which included specific enrichment in metabolism of polyketides and tetracyclic and pentacyclic triterpenes (cholesterol, steroids, and hopanoids) metabolism as the most enriched categories (Fig. 3c; see Tables S5 and S6 at https://doi.org/10.6084/m9.figshare.32975738). This suggests active secondary metabolite biosynthesis, which may play roles in fungal defense and environmental adaptation (Fig. 3c; see Tables S5 and S6 at https://doi.org/10.6084/m9.figshare.32975738). Virulence and disease factors were significantly enriched, indicating a potential enhancement of pathogenic traits under azole pressure (Fig. 3e; see Table S3 at https://doi.org/10.6084/m9.figshare.32975738). Concurrently, detoxification and transport ATPases were upregulated, pointing to mechanisms for toxin efflux and cellular cleansing (Fig. 3c; see Tables S5 and 6 at https://doi.org/10.6084/m9.figshare.32975738).

When we focused our analysis on DEGs identified in the *cyp51*-independent strains, we noted that, among the 1,397 unique downregulated genes, there was a pronounced enrichment in genes involved in cellular signaling, particularly genes involved in the MAPKKK cascade, modification by phosphorylation/dephosphorylation, and regulation of C-compound and carbohydrate metabolism (Fig. 3c; see Tables S5 and S6 at https://doi.org/10.6084/m9.figshare.32975738). Metabolic adaptations were also central, with significant enrichment in peroxisomal function and fatty acid metabolism (Fig. 3c; see Tables S5 and S6 at https://doi.org/10.6084/m9.figshare.32975738). Categorization of the 1,195 unique upregulated genes in the *cyp51*-independent strains revealed the most pronounced enrichment for processes related to energy metabolism and mitochondrial function. This is highlighted by strong upregulation in pathways such as aerobic respiration, mitochondrial transport, electron transport, and membrane-associated energy conservation and respiration (Fig. 3c; see Tables S5 and S6 at https://doi.org/10.6084/m9.figshare.32975738). This suggests a substantial metabolic rewiring, potentially supporting energy-demanding efflux or stress-response mechanisms in the absence of *cyp51* mutations. Furthermore, pathways linked to protein synthesis, folding, and quality control were notably enriched (Fig. 3c; see Tables S5 and S6 at https://doi.org/10.6084/m9.figshare.32975738). Enrichment analysis of the unique downregulated genes in the *cyp51*-dependent strains showed the most pronounced enrichment for specific transport and biosynthetic processes, including organic anion transport and metabolism of isoleucine (Fig. 3c; see Tables S5 and S6 at https://doi.org/10.6084/m9.figshare.32975738). Translation elongation was enriched, suggesting a decrease in protein synthesis (Fig. 3c; see Tables S5 and S6 at https://doi.org/10.6084/m9.figshare.32975738).

To demonstrate their direct involvement in azole resistance, we deleted selected genes that showed high levels of down- and upregulation in the presence of VOR, as observed in the RNA-seq experiments using the A1160 *pyrG*^+^ azole-susceptible background (see Tables S3 and S4 at https://doi.org/10.6084/m9.figshare.32975738). We chose: (i) AFUA_8G06610, encoding a major facilitator superfamily (MFS) transporter (shown as down- and upregulated in *CYP51*-independent strains); (ii) AFUA_1G12620, encoding a MFS transporter (shown as upregulated in *CYP51-*independent strains); (iii) AFUA_3G03300, encoding a FAD-binding protein (shown as down- and upregulated in *CYP51*-independent strains); (iv) AFUA_7G00710, encoding a MFS transporter (shown as upregulated in *CYP51*-dependent and *CYP51*-independent strains); (v) AFUA_1G01180, encoding a FAD-binding protein (shown as upregulated in *CYP51*-dependent and *CYP51*-independent strains); (vi) AFUA_5G06070, encoding *MDR1*, an ATP-binding cassette (ABC) transporter (shown as upregulated in *CYP51*-dependent and *CYP51*-independent strains); (vii) AFUA_8G04050, encoding the transcription factor XprG (shown as downregulated in *CYP51*-independent strain); (viii) AFUA_2G09890, encoding the transcription factor Ndt80 (shown as down- and upregulated in *CYP51*-independent strains); and (ix) AFUA_2G08370, a membrane-associated protein in eicosanoid and glutathione (MAPEG; shown as upregulated in *CYP51*-dependent and *CYP51*-independent strains). None of the null mutants showed differences in VOR susceptibility, suggesting that they are not involved in VOR resistance or are important only in the specific genetic background of the corresponding clinical isolate.

Recently, we identified 468 mitochondrion- and nucleus-encoded genes in *A. fumigatus* (20). When examining these specific transcripts, we noted that in *cyp51*-independent strains, about 89% of mitochondrial genes were significantly modulated (in contrast to 38% of mitochondrial genes differentially expressed in the *cyp51*-dependent isolates) (Table 2). Among the genes modulated exclusively for each phenotype, unique *cyp51-*independent down- and upregulated genes accounted for about 33% of mitochondrial genes, and unique *cyp51*-dependent genes accounted for about 1% (Table 2). These results highlight the significance of mitochondrion-encoded genes in *cyp51-*independent azole resistance.

**TABLE 2:** Number of mitochondrial nuclear-encoding genes in the CYP51-dependent and CYP51-independent strains

Treatment	Total DEG	Mitochondrial genes	Mitochondrial genes (%)
Cy51-dependent down	1,681	128	27.35
Cy51-dependent up	1,945	50	10.68
CYP51-independent down	4,240	127	27.14
Cyp51-independent up	3,947	290	61.97
Unique CYP51-dependent down	67	5	1.07
Unique CYP51-independent down	1,356	41	8.76
Unique CYP51-dependent up	99	0	0.00
Unique CYP51-independent up	1,195	114	24.36

### Resistance-associated variants, coupled with transcriptional profiling, reveal that *cyp51-*independent azole resistance is governed by complex circuitry

To further identify loci contributing to both canonical and non-canonical resistance phenotypes, we performed treeWAS analysis, a phylogenetically informed GWAS approach. Using a significance threshold of *P* < 0.01, the analysis revealed that *cyp51*-dependent resistance is associated with many more significant SNPs (3,534 SNPs) spread across many genes (910) compared to the *cyp51-*independent phenotype (1,372 SNPs in 990 genes) (see Fig. S2; Table S7 at https://doi.org/10.6084/m9.figshare.32975738), likely reflecting the stronger selective sweep signatures associated with target site mutations in clade A. Despite the different primary mechanisms, a core set of 116 non-synonymous genes and one intergenic region (AFUA_4G09080-AFUA_4G09090) harbor variants associated with both resistance types.

The *cyp51*-independent 1,172 non-synonymous SNPs (from the total 1,372) were cross-referenced with transcriptomic results to identify pathways important for mediating non-canonical azole resistance (see Table S8 at https://doi.org/10.6084/m9.figshare.32975738). The most significant associations included a protein kinase with multiple mutations (AFUA_2G14920; repressed), isoamyl alcohol oxidase (AFUA_1G01180; sharply overexpressed, in contrast to no change in the cyp51-dependent group), an MFS transporter (AFUA_8G04470; overexpressed), and the GTP-binding protein EsdC (AFUA_7G01930; repressed). Interestingly, some significant SNPs were identified in genes that were not differentially expressed, including an MFS transporter (AFUA_5G10340) with multiple mutations and an ABC drug exporter, AbcA (AFUA_1G17440). Looking at major categories, a collection of transport and ribosomal synthesis genes was highly associated, encompassing 73 non-synonymous mutations in transport genes and 22 ribosomal synthesis genes.

**Fig 4:**
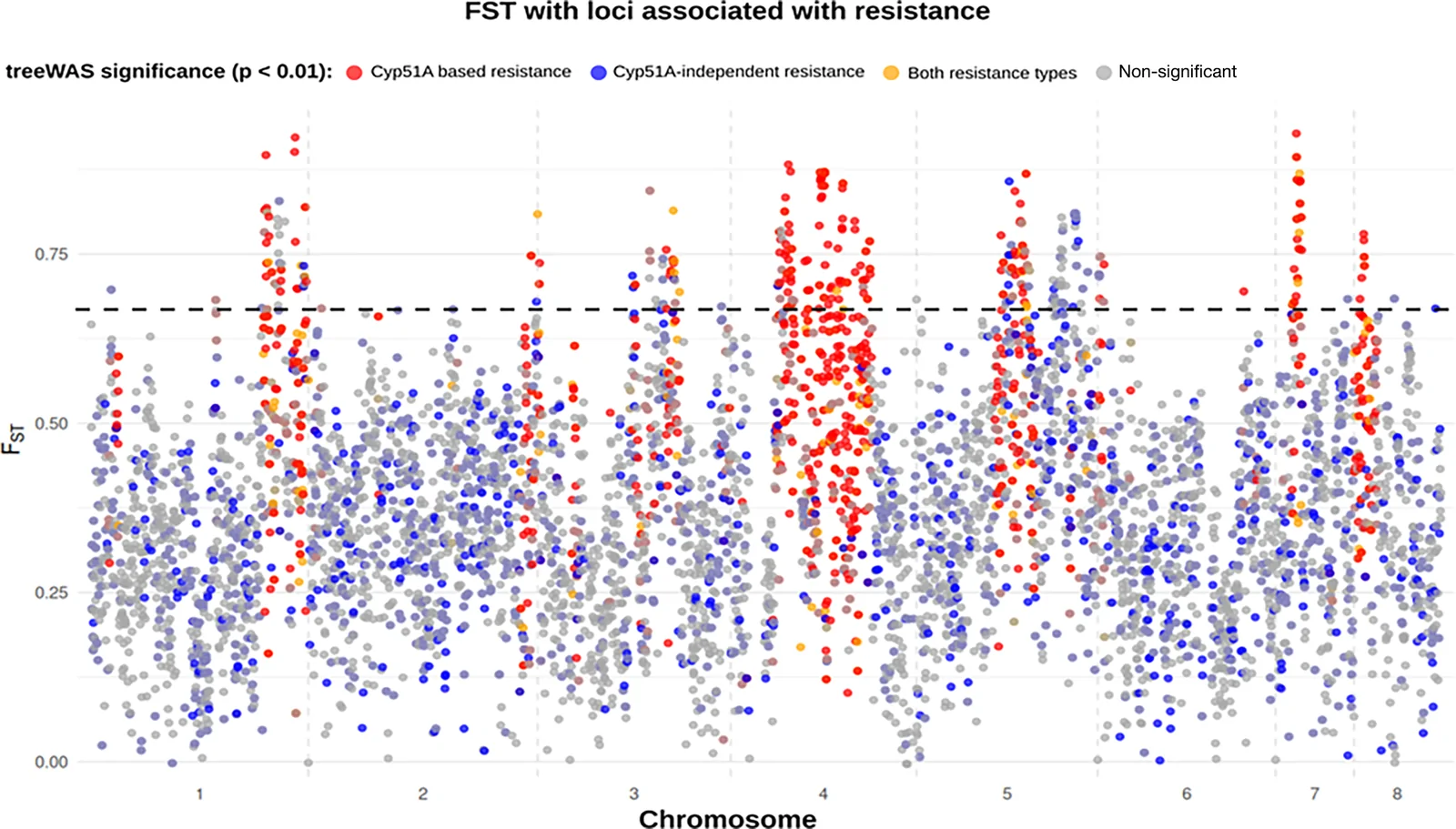
The fixation index (FST) analysis coupled with treeWAS significance. Scatterplot of sliding 5-kb non-overlapping window estimates of FST for each chromosome between isolates within clades A and B. In addition, the dots representing the genomic windows were painted according to the presence of mutations significantly associated with azole resistance.

Finally, mapping significant treeWAS SNPs onto FST Manhattan plots (comparing clade A vs clade B) (Fig. 4) revealed a non-random spatial distribution of selection. *cyp51*-dependent SNPs (red dots) were almost exclusively concentrated in the highest FST peaks, appearing in dense clusters. This supports that canonical resistance in clade A results from strong directional selection acting on specific chromosomal loci, leading to localized genomic hitchhiking and the formation of high-differentiation blocks. In contrast, *cyp51*-independent SNPs (blue dots) are largely absent from the highest peaks and instead are distributed across regions of lower differentiation. These results are consistent with a polygenic architecture underlying *cyp51*-independent resistance that integrates transcriptional, post-transcriptional, and signaling networks across azoles.

### Transcriptomic signatures of the major clades background

**Fig 5:**
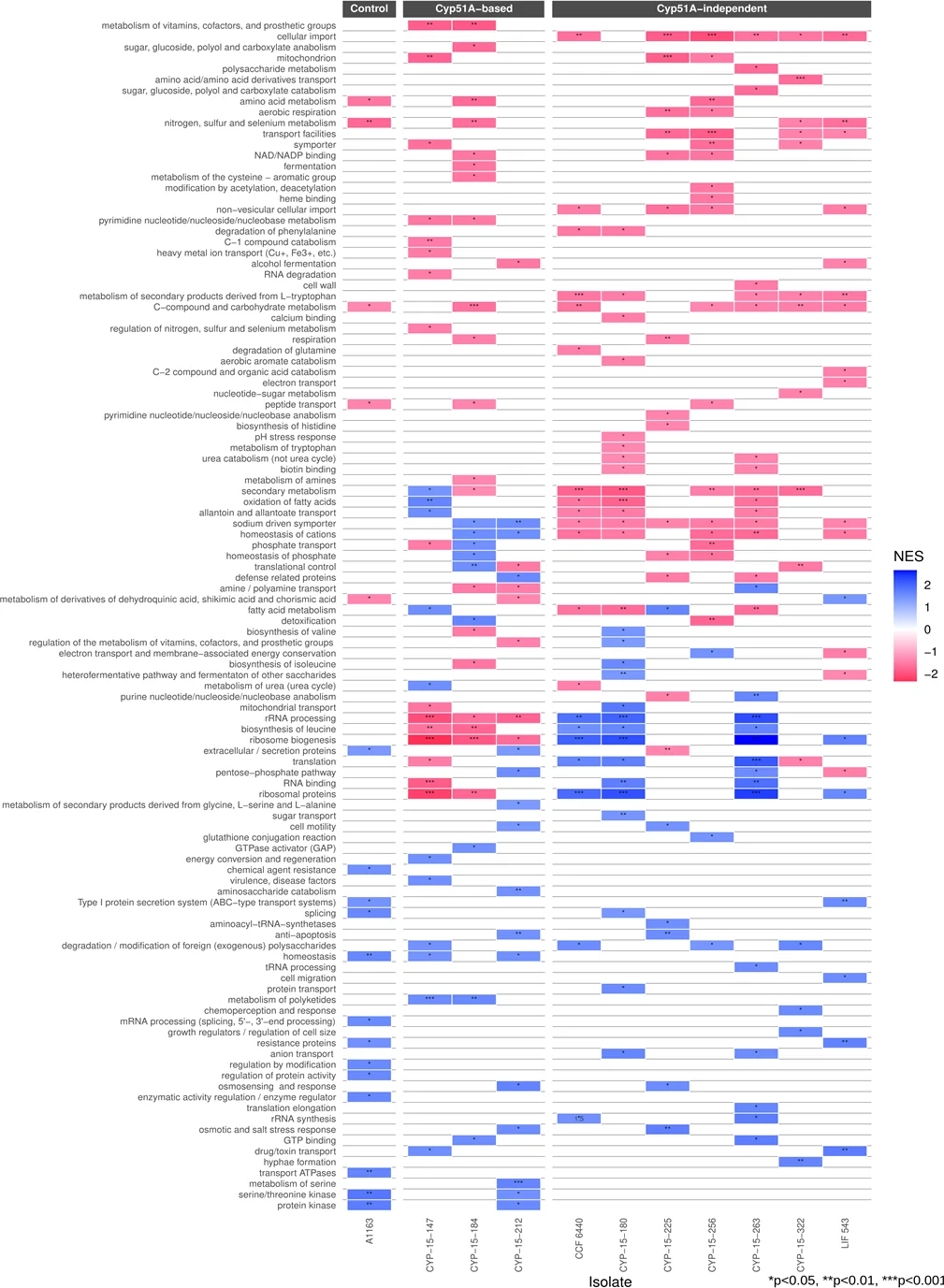
Heatmap of gene set enrichment analysis (GSEA) applied to genes within the 90th percentile of FSTs windows identified for canonical versus non-canonical resistance.

To study the regulatory patterns associated with the genetic background from clade differentiation, gene set enrichment analysis (GSEA) was applied to genes within the 90th percentile of FST windows identified (clade A vs clade B), combined with gene fold change results (RNAseq under azole). The categorization of GSEA results (Fig. 5) showed a few patterns: *cyp51*-independent resistance was downregulated for cellular import, transport facilities, non-vesicular cellular import, C-compound and carbohydrate metabolism, metabolism of secondary products derived from L-tryptophan, secondary metabolism, sodium-driven symporter (upregulated for *cyp51* dependent), and homeostasis of cations (upregulated for *cyp51* dependent). Meanwhile, *cyp51*-dependent resistance is downregulated for rRNA processing, leucine biosynthesis, ribosome biogenesis, and ribosomal proteins, while these four categories are upregulated for *cyp51* independent. Overall, these findings reveal novel genetic variants and unique population structures between *cyp51-*dependent and *cyp51*-independent isolates, advancing our understanding of the evolutionary dynamics of resistance in *A. fumigatus*.

### Functional enrichment of azole-exclusive eQTLs

**Fig 6:**
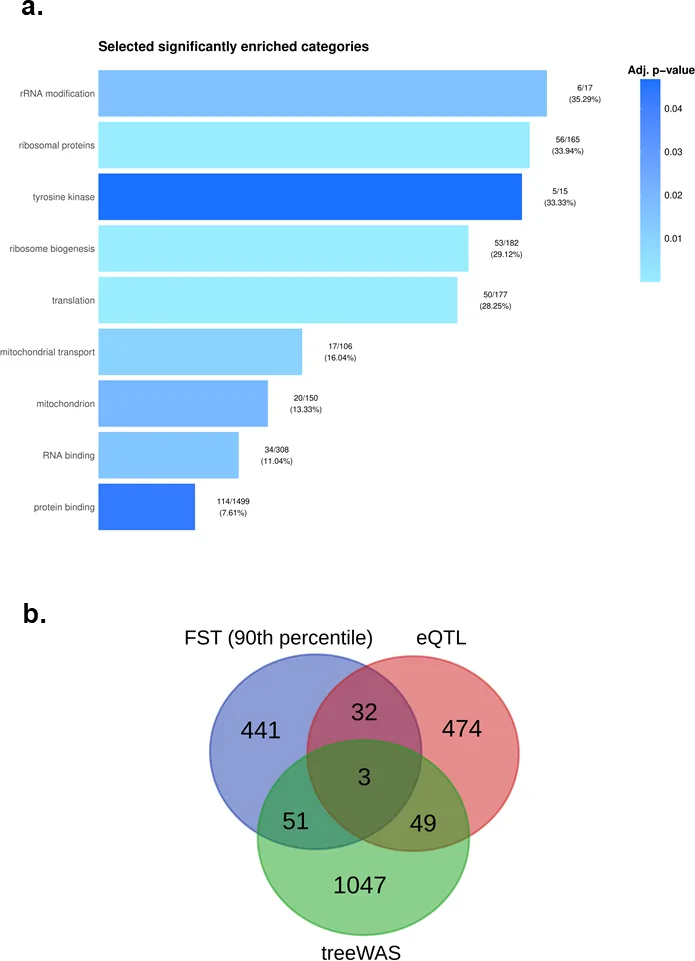
Functional enrichment and multi-omic integration of azole-responsive regulatory variants. (**a**) Enrichment analysis of genes harboring eQTLs exclusive to azole treatment in non-canonical resistant isolates. A total of 3,964 SNPs (mapping to 558 genes) were mapped against FunCat categories using FungiFun3, with significance assessed by Fisher’s exact test and Bonferroni correction (*P* < 0.05). (**b**) Venn diagram intersecting these azole-exclusive eQTL target genes with the top 10% FST windows separating canonical from non-canonical resistant groups and treeWAS-identified variants associated with the non-canonical resistance phenotype.

To characterize the regulatory response of non-canonical resistant isolates under drug stress, we identified 3,964 significant eQTLs exclusive to the azole treatment, mapping to 558 unique genes. FunCat enrichment analysis showed a strong overrepresentation of the translational machinery, including ribosome biogenesis, rRNA modification, ribosomal proteins, and translation (Fig. 6a; see Table S9 at https://doi.org/10.6084/m9.figshare.32975738). Additionally, genes involved in mitochondrial transport, mitochondrion function, tyrosine kinase activity, and RNA/protein binding were significantly enriched.

We intersected the azole-exclusive eQTL-regulated genes with genomic regions under high selection (top 10% FST) identified by comparing canonical and non-canonical resistant isolates, as well as with variants associated with non-canonical resistance via treeWAS. This intersection revealed three high-confidence candidates: a tetratricopeptide repeat protein (AFUA_8G00350), the 40S ribosomal protein uS5 (AFUA_7G01460), and the putative AIF-like mitochondrial oxidoreductase Nfrl (AFUA_7G02070) (Fig. 6b; see Table S9 at https://doi.org/10.6084/m9.figshare.32975738).

## DISCUSSION

The findings indicate that cyp51-independent azole resistance in *A. fumigatus* arises from diverse molecular adaptations rather than a single dominant mechanism. The recurrent detection of transcription factors and RNA metabolism genes across ITC-, VOR-, and POS-resistant isolates suggests that transcriptional reprogramming and post-transcriptional control are central to the evolution of resistance. Recent work has identified transcriptional regulators, such as HbxA and C_2_H_2_-type zinc finger proteins, as modulators of azole stress responses via calcium–calcineurin signaling and chromatin remodeling pathways (21). This regulatory rewiring resembles global stress adaptation mechanisms observed during antifungal exposure, wherein transcriptional flexibility enhances fungal survival under chemical stress.

The involvement of cytoskeletal and cell wall biosynthesis genes, including *Ags2* (α-1,3-glucan synthase) and *ChsE* (chitin synthase), suggests that *A. fumigatus* strengthens structural integrity to limit azole entry. Although this hypothesis requires validation, altered wall composition and glucan–chitin balance have been correlated with reduced azole permeability and enhanced tolerance in clinical isolates (22). For VOR and POS, the emergence of chromatin- and signaling-related components (e.g., HosB, SakA, and calcineurin subunits) in resistant isolates supports a model wherein epigenetic plasticity and stress signaling modulate resistance phenotypes. This aligns with previous evidence that the calcineurin pathway contributes to azole tolerance by modulating cell wall integrity and stress responses (21). The extensive activation of antioxidant defenses and efflux systems in POS-resistant isolates further underscores the multifactorial and drug-specific nature of *cyp51*-independent resistance. Collectively, these data suggest that *A. fumigatus* evolves complex, multilayered defense networks to withstand azole pressure, emphasizing the need for combination therapies targeting stress signaling, transcriptional regulation, and ergosterol biosynthesis.

Functional enrichment analysis of RNA-seq data from a VOR-susceptible strain (A1160 *pyrG*+) and two resistant groups with distinct mechanisms (*cyp51*-dependent and *cyp51*-independent) revealed fundamentally different transcriptional landscapes. Cyp51-dependent strains exhibited a classic stress-response phenotype, characterized by significant upregulation of catabolic, import, and detoxification pathways (allantoin transport, non-vesicular cellular import, and secondary metabolism), alongside marked downregulation of the protein synthesis machinery (ribosome biogenesis, rRNA processing, and translation). This pattern suggests resource reallocation, in which the cell dampens costly anabolic processes to fuel detoxification and nutrient scavenging, which are critical for survival under azole stress. Conversely, *cyp51*-independent strains displayed a strikingly opposite profile, with robust upregulation of biosynthetic and energetic capacity—enrichment in ribosomal proteins, translation, mitochondrial function, and mitochondrial transport—and strong downregulation of broad metabolic functions (lipid, fatty acid, and nitrogen metabolism). This indicates a strategy of enhancing core cellular production and energy generation while streamlining peripheral metabolic pathways.

The VOR-susceptible clinical isolate (A1160 *pyrG*+) showed a more muted, targeted response than the resistant strains, with upregulated genes associated with stress defense and cell wall maintenance (detoxification, virulence factors, and polysaccharide metabolism) and downregulated genes involved in sulfate assimilation, chitin metabolism, and cellular import. In conclusion, the three strain types employ divergent survival strategies: *cyp51*-dependent strains prioritize stress defense over growth, *cyp51*-independent strains enhance growth and energetic machinery, and the susceptible A1160 *pyrG*+ strain mounts a limited defensive response primarily involving detoxification and cell wall remodeling. These findings highlight that the mechanism of azole resistance dictates fundamental transcriptional reprogramming.

The transcriptomic signature of the top FST windows in *cyp51*-independent isolates (clade B) is characterized by metabolic repression and translational investment. Downregulation of cellular import, transport, and metabolic pathways (carbohydrate and secondary products of L-tryptophan) suggests a response aimed at reducing intracellular azole accumulation by restricting the activity of nonessential symporters and transporters. This is coupled with paradoxical upregulation of ribosome biogenesis, rRNA processing, and ribosomal proteins. Ribosome production, one of the most energy-demanding cellular processes, typically coupled to rapid growth and high nutrient availability (23), is prioritized under azole stress in clade B isolates, suggesting preparation for massive protein synthesis that could favor the production of high-level efflux systems (24). In *A. fumigatus*, ribosome biogenesis is governed by factors such as *cgrA* (25), which is essential for rapid growth at host body temperature and contributes significantly to virulence. Conversely, *cyp51*-dependent isolates (clade A) downregulate ribosome biogenesis and primary metabolic pathways (e.g., leucine biosynthesis) while upregulating cation homeostasis and symporters, suggesting that clade A isolates with high-efficiency target site mechanisms (*cyp51* mutations) may not require broad metabolic shifts but instead focus on managing physiological perturbations from altered sterol composition, such as maintaining electrochemical gradients across modified membranes.

The strong genome-wide differentiation between clades A and B indicates that azole resistance in *A. fumigatus* has evolved atop a deep and persistent population structure rather than emerging solely through recent selective events. The concentration of high FST windows between clades, coupled with consistently negative Tajima’s *D* values in both lineages, supports a model wherein long-standing divergence has been repeatedly reinforced by contemporary selection. Such patterns are characteristic of genomic regions under recurrent or ongoing positive selection, even when the initial split predates the selective agent itself (26, 27). In *A. fumigatus*, environmental exposure to agricultural azoles has shaped the evolution of resistance over decades, acting on pre-existing genetic backgrounds (11, 17). The overlap between extreme FST peaks and negative Tajima’s *D* values suggests that clade-defining loci remain targets of selection, likely because they modulate fitness under azole stress and other environmental pressures.

A key outcome is the contrast between genetic architectures underlying *cyp51*-dependent and *cyp51*-independent resistance. Canonical resistance in clade A is characterized by clustered, high-effect variants within regions of extreme differentiation, consistent with hard selective sweeps driven by target site mutations and strong genomic hitchhiking (28). In contrast, *cyp51*-independent resistance within clade B shows low differentiation from susceptible isolates and a diffuse genomic signal, with associated SNPs spread across many loci of modest effect, a pattern consistent with polygenic adaptation acting on standing genetic variation, which is increasingly recognized in fungal drug resistance and microbial adaptation (29). Variant enrichment in transporters, signaling components, and ribosomal biogenesis genes, together with discordant transcriptional regulation, implies that resistance can arise through coordinated modulation of cellular physiology rather than single-target disruption. However, a small number of independent-associated SNPs appear at high peaks, suggesting that while non-canonical resistance is broadly polygenic, it may occasionally leverage high-differentiation alleles that define the clades. Formal genetic crosses would be required to definitively prove polygenicity.

Enrichment of ribosome biogenesis and translation categories among azole-exclusive eQTLs reinforces the distinct adaptive strategy of clade B isolates. Unlike canonical resistant isolates, which downregulate energy-intensive protein synthesis to fuel detoxification, at least some non-canonical resistant isolates prioritize translational investment. Ribosome production is essential for rapid growth (23) and, in *A. fumigatus*, is governed by factors like *cgrA*, which is critical for virulence (25). This strategy likely supports the synthesis of complex defense networks, such as high-level efflux systems, required for survival without target site mutations (24). Furthermore, modulation of nuclear-encoded mitochondrial genes aligns with the metabolic rewiring observed in non-canonical resistance, in which mitochondrial energy generation and transport are prioritized (20).

The convergence of eQTL, FST, and treeWAS signals on AFUA_8G00350, AFUA_7G01460, and AFUA_7G02070 identifies these genes as core nodes in the polygenic architecture of clade B. AFUA_7G02070 (NfrI), an AIF-like mitochondrial oxidoreductase, underscores the importance of mitochondrial redox balance and metabolic stability under azole pressure (20, 20). Similarly, selection of the ribosomal protein AFUA_7G01460 (23) and the tetratricopeptide repeat (TPR) protein AFUA_8G00350 (a structural mediator of protein-protein interactions) suggests that regulatory variation in the translational apparatus and protein interaction networks provides plasticity required for non-canonical resistance (29). Together, these findings demonstrate that cyp51-independent resistance relies on a multilayered network of metabolic and translational adaptations rather than single target site alterations (13, 24).

Pangenome-wide associations highlight the importance of accessory genes and gene clusters in shaping non-canonical resistance phenotypes. Accessory genomes in filamentous fungi are enriched for niche-adaptive functions, including secondary metabolism and xenobiotic detoxification, and vary substantially among lineages (30, 31). The association of clustered genes, such as the *cyp52* region, with mitochondrial carriers like Pet8 suggests that metabolic rewiring and membrane-associated processes contribute to azole tolerance in clade B. The “CYP52 cluster” (cluster 33 [32]) occurs in a small subset of the population (32/370 isolates), yet is highly enriched in *cyp51*-independent resistant strains (26/32) and is almost absent from clade A (1/32). Hopanoids are pentacyclic molecules present in the membranes of some bacteria and in *S. japonicus*, where they have been proposed as sterol surrogates in the latter organism under anoxic conditions (33–35). However, cluster 33, potentially involved in hopanoid biosynthesis, has not yet been characterized, and its role in non-canonical azole resistance requires validation. This suggests that the CYP52 cluster may represent a specialized metabolic module conferring a survival advantage under azole stress, potentially by stabilizing fungal membranes through hopanoid production or facilitating azole detoxification (36, 37).

We note that the data set is enriched with isolates from the United Kingdom, reflecting the availability of public data. However, the strong phylogenetic signal (clade A vs B) was independent of geographic origin. Future studies with more balanced global sampling will be necessary to assess additional lineage-specific resistance mechanisms in under-sampled regions. A limitation is the incomplete separation of clade background from resistance mechanism, as 65/66 non-canonical resistant isolates fall within clade B. While our phylogenetic, transcriptomic, and accessory genome data suggest that clade B harbors a genetic architecture permissive for polygenic azole resistance, future studies with larger, balanced collections are needed for within-clade GWAS and formal estimation of recombination rates.

The strong enrichment of non-azole fungicide resistance mutations (e.g., in *benA* and *sdhB*) within clade A, tightly associated with *cyp51A* tandem repeat alleles, provides additional evidence that canonical azole resistance in this lineage is frequently linked to environmental fungicide exposure. Conversely, the complete absence of such markers among *cyp51*-independent resistant isolates (clade B) argues against an environmental origin for non-canonical resistance. This pattern aligns with the proposal that *cyp51A*-independent azole resistance arises primarily in clinical settings, such as during prolonged azole therapy, rather than from agricultural selection pressure. However, we cannot formally rule out untested environmental drivers or different fungicide classes that select for non-canonical resistance pathways. Future experimental evolution studies that directly compare clinical and environmental isolates will be required to resolve the origin of this type of resistance.

Together, these findings support a model in which *cyp51*-independent resistance arises from the combined effects of polygenic SNP variation, transcriptional reprogramming, and accessory gene content, providing a flexible evolutionary route that may facilitate rapid adaptation without the fitness costs often associated with canonical target site mutations.

## MATERIALS AND METHODS

### Origin of fungal isolates and culture medium

The clinical isolates of *Aspergillus fumigatus* shown in Table 1 are deposited in the Molecular Biology Laboratory of the Faculty of Pharmaceutical Sciences of Ribeirão Preto, University of São Paulo. The strains were cultured and maintained on solid or liquid minimal medium at 37°C (liquid minimal medium: 1% [wt/vol] glucose, 50 mL of 20× saline solution, trace elements, 2% [wt/vol], pH 6.5; the solid minimal medium contains the same components but with the addition of 2% agar). The trace element and saline solutions are described by Käfer (38).

### Minimum inhibitory concentration (MIC) determination assay

The MIC was visually determined after a 48-hour incubation at 37°C, in accordance with the guidelines established by the Clinical and Laboratory Standards Institute (M38-A3) (39). The azoles itraconazole, voriconazole, and posaconazole were tested at concentrations ranging from 0 to 4 µg/mL. Briefly, MIC assays were conducted in RPMI-1640 medium or minimal medium (MM) using flat-bottom 96-well polystyrene microplates and an initial inoculum of 2.5 × 10⁴ conidia/mL. The MICs for itraconazole, voriconazole, and posaconazole were defined as the lowest concentration of each drug that resulted in 100% growth inhibition relative to the drug-free control, as determined visually after 48 h of incubation at 37°C without agitation. The *A. fumigatus* A1160 *pyrG*^+^ strain was used as a control. Interpretation of the MIC values was performed in accordance with CLSI M38M51S (40). Isolates were categorized as resistant when MICs were as follows: VOR ≥ 2 mg/L, ISA ≥ 4 (or ≥ 2 mg/L if VOR resistant), ITRA ≥ 2 mg/L, and POS ≥ 0.5 mg/L.

### RNA extraction, purification, and sequencing preparation

A total concentration of 10⁶ spores/mL of clinical *A. fumigatus* isolates was inoculated into three biological repetitions of 50 mL of liquid minimal medium and incubated at 37°C for 24 h with agitation in the absence or presence of VOR at 0.5× MIC. Total RNA was extracted using TRIzol, treated with RQ1 RNase-free DNase I (Promega), and purified using the RNeasy kit (Qiagen), according to the manufacturer’s instructions.

Total RNA quantification was performed using a NanoDrop, and RNA integrity was analyzed using an Agilent 2100 Bioanalyzer. All RNA samples had a minimum RNA integrity number (RIN) value of 8.0. For RNA sequencing, cDNA libraries were constructed using the TruSeq Total RNA with Ribo Zero kit (Illumina, San Diego, USA). Starting from 0.1 to 1 µg of total RNA, ribosomal RNA was removed, and the remaining RNA was purified, fragmented, and prepared for complementary DNA (cDNA) synthesis, following the manufacturer’s recommendations. Libraries were validated according to the Library Quantitative PCR (qPCR) Quantification Guide (Illumina).

### DNA extraction from isolates

DNA extraction was performed using the DNeasy UltraClean Microbial Kit (Qiagen, Hilden, Germany) according to the manufacturer’s protocol. The initial fungal material was obtained using a moistened sterile swab to collect spores, which were subsequently dissolved in 700 μL of sterile saline solution. The suspension was then centrifuged at 10,000 rpm for 4 min. The supernatant was discarded, and the pellet was resuspended in 300 μL of Power Bead Solution. The final concentrated suspension was transferred to a Pathogen Lysis Tube L (Qiagen, Hilden, Germany) containing beads, 50 μL of SL solution, and 200 μL of sterile saline solution, which was added for homogenization. Cell disruption was performed using a Precellys 24 tissue homogenizer (Bertin, Montigny-le-Bretonneux, France), programmed for three 30-second cycles at 5,000 rpm with 30-second intervals between cycles. The isolated DNA was then sent to a specialized facility for WGS library preparation.

### Acquisition of publicly available data

The data set for this project includes genomic information from publicly accessible sequencing projects involving *A. fumigatus* isolates that were previously classified as resistant or susceptible. Pre-selected strains are part of the selected studies (NCBI accession numbers: PRJEB27135, PRJDB10244, PRJEB8623, PRJNA638646, PRJNA798608, PRJNA592352, PRJNA548244, PRJNA961646, and PRJNA592352), which provided descriptions of isolate resistance or susceptibility through *in vitro* assays, totaling 334 complete genomic sequences. Additionally, 36 *A. fumigatus* isolates were sequenced and had their MIC previously determined as described above. In total, the 370 isolates analyzed consisted of 191 azole-resistant isolates and 179 azole-sensitive isolates. Of these, 66 resistant isolates lack mutations in the *cyp51A* gene presumed to confer azole resistance, suggesting a non-canonical mechanism of resistance. The sequences were obtained from GenBank (41) using the SRA Toolkit v3.0.2 (available at github.com/ncbi/sra-tools).

### Genome assembly and annotation

Sequence quality was assessed using FastQC v0.11.8 (42). Adapter sequences were removed, and reads were quality-filtered using Trimmomatic v0.39 (43), with the parameters “ILLUMINACLIP:NexteraPE-PE.fa:2:30:10 LEADING:20 TRAILING:20 MINLEN:60.” *De novo* genome assemblies were performed using SPAdes v3.14.0 (44). Genome completeness was inferred with BUSCO v5.4.4 (45) using eurotiales_odb10 as the lineage parameter. Genomes were annotated through automatic protein prediction with AUGUSTUS v3.5.0 (46), using the parameter “--species=aspergillus_fumigatus.”

### Variant identification and genome-wide association study (GWAS)

To detect new variants, the processed reads were mapped to the *A. fumigatus* Af293 reference genome (NCBI assembly code GCA_000002655.1) using Bowtie v2.3.5.1 (47). The generated SAM files were converted to BAM format using SAMtools v1.15.1 (48). Subsequently, single-nucleotide polymorphisms (SNPs) were identified using the Genome Analysis Toolkit (GATK) v4.3.0.0 (49) with the “HaplotypeCaller” module. All the variants in VCF files were combined using the “CombineGVCFs” module to create a single file for genotyping with “GenotypeGVCFs.” Low-confidence SNPs were filtered using “VariantFiltration” with the parameters “QualByDepth < 25.0 || MappingQuality < 55.0 || MappingQualityRankSumTest < −0.5 || FisherStrand > 5.0 || StrandOddsRatio > 2.5 || ReadPosRankSumTest < −2.0.”

For phylogenetic analysis, the merged VCF file was converted to PHYLIP format using vcf2phylip v2.0 (available at https://zenodo.org/record/2540861). High-confidence SNPs were used for the inference of the maximum-likelihood tree using IQ-TREE v1.7 (50) with default parameters. The substitution model TVM + F + G4 was automatically selected, and the ascertainment bias correction (+ASC) was applied. The generated tree was edited in iTOL v6 (51).

To identify loci associated with azole resistance, we performed a genome-wide association analysis using treeWAS (52), a method that employs phylogenetic information to correct for microbial population structure. The maximum-likelihood phylogenetic tree, previously inferred from genome-wide SNPs using IQ-TREE, was used as input. To perform the treeWAS analysis, azole resistance was treated as a binary phenotype, considering isolates as either susceptible or resistant. The analysis was conducted using the treeWAS() function with default parameters and performed separately for canonical and non-canonical resistance. Among the statistical tests performed by default (terminal, simultaneous, and subsequent), only the results of the subsequent test were considered based on the recommendation by Collin and Didelot that the subsequent test is the most effective subtle patterns of association (52). A raw *P*-value cutoff of 0.01 was applied. Manhattan plots with *P*-value distribution were created using the R package qqman (53) for canonical and non-canonical resistance-associated SNPs.

Population genetic statistics Fst and Tajima’s *D* based on the SNPs were calculated with VCFtools v0.1.16 (54) using a non-overlapping 5 kb window. The results were plotted with the R package qqman (53) and ggplot2 (55).

### Pangenome analysis and accessory gene associations

To investigate gene content variation across the *A. fumigatus* isolates, a pangenome analysis was conducted using the complete set of 370 genomes included in this study. Orthologous gene groups were inferred using OrthoFinder (v2.5.5) (56). Protein sequences predicted from each genome assembly were used as input for the analysis. All-versus-all sequence similarity searches were performed with DIAMOND in BLASTP mode to identify homologous protein sequences across genomes (57). The orthogroup matrix generated by OrthoFinder was subsequently used to test for associations between gene presence–absence patterns and azole resistance phenotypes, which were tested using SCOARY v1.6.16 (58). SCOARY performs a microbial pangenome-wide association analysis by testing for statistical associations between binary gene presence–absence data and phenotypic traits. The phenotypic traits considered for SCOARY were clade A vs clade B, canonical vs susceptible, and non-canonical vs susceptible. Statistical significance was assessed using Fisher’s exact test with multiple-testing correction as implemented in SCOARY.

### Differential expression analysis

Sequence quality was assessed using FastQC v0.11.8 (42). Adapter sequences were removed, and reads were quality-filtered using Trimmomatic v0.39 (43) with the parameters “ILLUMINACLIP:NexteraPE-PE.fa:2:30:10 LEADING:20 TRAILING:20 MINLEN:60.” The filtered reads were aligned to the reference sequences of *A. fumigatus* Af293 (accession code GCA_000002655.1) and *A. fischeri* NRRL 181 (accession code GCF_000149645.3) using Hisat v2.2.1 (59), with splice site positions and maximum intron length, based on the Af293 (*A. fumigatus*). Subsequently, SAM files were converted to BAM format and sorted using SAMtools v1.15.1 (54). FeatureCounts v1.5.3 (60) was used to quantify unambiguous aligned fragments and produce tables for statistical analysis of differential expression. For the differential expression analysis, counts were converted to counts per million (CPM). Features were filtered to ensure that the sum across all samples was at least 3 CPM, considering only samples with a CPM above 1. Differential expression analysis of the filtered genes was performed using edgeR v3.14.0 (61) with the “simple design protocol” (62).

### Deletion by CRISPR/Cas9 technology

CRISPR/Cas9 vectors were constructed as previously described (63) using primers (see Fig. S3; Table S10 at https://doi.org/10.6084/m9.figshare.32975738) designed based on the *A. fumigatus* genome (obtained from https://fungidb.org). To generate each fragment for vector construction, we amplified small DNA fragments by PCR using a DNA polymerase Pfu X7 and the vector pFC902 as a template. Amplified fragments were treated using DpnI (New England Biolabs) and then purified using a 2% agarose gel. After purification, PCR fragments were cloned using USER cloning (64) into the vector pFC330 (CRISPR/Cas9), which had been previously digested with the PacI (NEB) and Nt.BbvcI (NEB) enzymes, and transformed into *E. coli* DH5α. Transformation using *A. fumigatus* Δ*ku* A1160 *pyrG*^−^ strain protoplasts (65) utilized the vectors and synthesized repair oligonucleotides for homologous recombination, composed of upstream and downstream sequences of the target genes (see Table S10 at https://doi.org/10.6084/m9.figshare.32975738). Transformants growing without uracil and uridine were isolated and subjected to five rounds of monosporic purification. Deletions were confirmed by diagnostic PCR.

### Gene set enrichment analysis (GSEA)

To identify enriched functions among highly differentiated genes, we applied gene set enrichment analysis (GSEA) to genes in the top 90th percentile of FST windows between clade A and clade B, combined with each respective gene fold-change data for the azole vs minimum media conditions for each tested isolate. This gene list was analyzed using FungiFun3 (19), a web-based tool for fungal gene set enrichment. We submitted the gene list as an unranked set in FungiFun3’s overrepresentation analysis mode, using the *A. fumigatus* Af293 annotation. FunCat categories were tested for overrepresentation. Enrichment significance was assessed by Fisher’s exact test with Benjamini–Hochberg correction (as implemented in FungiFun3); categories with FDR < 0.05 were reported as significantly enriched.

### eQTL mapping and integrative analysis

We performed eQTL mapping to identify genetic variants affecting gene expression. Genotypes were extracted from VCF files with bcftools v1.15.1 (48) and coded as 0 (reference) or 1 (alternate). Expression data (FPKM) were log₂(FPKM + 1)-transformed to stabilize variance. To account for population structure, we ran PCA on the genotype matrix (excluding monomorphic SNPs) using R’s prcomp function and included PC1 and PC2 as covariates in the linear model. Cis-eQTL mapping was performed with the Matrix eQTL R package (66) using a linear regression and a 10 kb window around each gene. The analysis was conducted separately for minimal medium and azole-treated conditions to capture condition-specific effects. Significance was defined by a Bonferroni-corrected *P*-value < 0.01. We overlapped the top regulated genes with FST-based selection signatures and treeWAS-identified resistance-associated SNPs. Finally, genes with eQTLs exclusive to azole treatment were tested for functional enrichment. We ran over-representation analysis (ORA) using the FungiFun3 platform (19), with FunCat categories and the Af293 genome as background. Significance was set at Bonferroni-corrected *P* < 0.05 (Fisher’s exact test).

## Data Availability

The WGS and RNA-sequencing data were submitted to NCBI under the BioProject ID PRJNA1439313.
